# Candesartan—the next anti-amyloid drug?

**DOI:** 10.1093/braincomms/fcac293

**Published:** 2022-11-07

**Authors:** Sevil Yasar

**Affiliations:** Division of Geriatric Medicine and Gerontology, Department of Medicine, Johns Hopkins School of Medicine, Baltimore, MD, USA

## Abstract

This scientific commentary refers to ‘Safety and biomarker effects of candesartan in non-hypertensive adults with prodromal Alzheimer's disease’ by Hajjar *et al.* (https://doi.org/10.1093/braincomms/fcac270).

**This scientific commentary refers to ‘Safety and biomarker effects of candesartan in non-hypertensive adults with prodromal Alzheimer's disease’ by Hajjar *et al.* (https://doi.org/10.1093/braincomms/fcac270).**

Alzheimer’s disease is a major, burdensome public health problem expected to increase due to the aging of the world population.^1^ Additionally, 15% of people aged 60 years and above carry a diagnosis of mild cognitive impairment (MCI), and these people are at 8–15% risk of developing dementia over 1 year.^2^ There are currently few modestly effective medications available to treat symptoms.^3^ There are no disease-modifying medications, and agents targeting amyloid β have failed in trials targeting Alzheimer’s disease and MCI.^4^ Without effective therapy for dementia, specifically Alzheimer’s disease, there will be an estimated three-fold increase in new Alzheimer’s disease cases, up to 13.2 million, by the year 2050 in the USA alone.^5^ Thus, identifying new and potentially effective approaches to prevention and/or treatment is critical.

With the lack of specific therapy for Alzheimer’s disease, there is increased interest in the repurposing of drugs, namely the use of drugs available and approved for other indications. Drug repurposing has been successfully applied in research areas and significantly shortens the time for potential drug development.

Alzheimer’s disease has been characterized by multiple processes, including amyloid β deposition, increased intracellular neurofibrillary tangles, oxidative stress, neuroinflammation, altered glucose metabolism, increased cerebral insulin resistance, dysregulation of calcium homeostasis, and mitochondria dysfunction, which leads to brain atrophy.^6^ The complex pathology opens the possibility of multiple targets for new therapeutic approaches.

There is emerging evidence for the involvement of the renin angiotensin system (RAS) in Alzheimer’s disease pathogenesis.^6,7^ Antihypertensive medications acting through the RAS, in addition to their blood pressure-lowering effect, could exert their effect on the pathogenesis of Alzheimer’s disease by various mechanisms, including antioxidant, anti-inflammatory, antithrombotic, anti-excitotoxicity, angiogenesis promoting effects, improvement of vascular endothelial function, or modulation of amyloid and tau metabolism.^6^ Medications acting via RAS could fulfill the criteria of potentially targeting multiple pathological processes in Alzheimer’s disease and could be a good candidate for development. However, meta-analyses of randomized controlled trials (RCTs) evaluating antihypertensive medication acting via RAS for prevention found no significant risk reduction of dementia.^8^ The lack of significant findings to date could be explained by dementia outcome being a secondary outcome; therefore, previous studies may not have been sufficiently powered to capture effects. Additionally, the loss of many participants to follow-up and a significant number of crossovers of participants from placebo to active treatment can influence results.^9^

To address this gap, there are currently numerous small ongoing RCTs using antihypertensive medications acting via RAS with cognitive function as an outcome in the hope of informing larger trials.^7^ The study by Hajjar *et al.*^10^ in *Brain Communications* is one of these; it is unique since it is the only study that involves participants with MCI and without hypertension. In this RCT, the authors evaluated the safety and efficacy of candesartan, an angiotensin 1 receptor blocker (AT1RB), and its effect over 1 year on cognition, cerebrospinal fluid (CSF) biomarkers (amyloid-β42, amyloid-β40, total tau and phosphorylated tau), PET imaging [Pittsburgh compound-B (^11^C-PiB) and ^18^F-flortaucipir], resting state functional brain MRI and domain-specific and global cognitive function in 77 normotensive participants with MCI. The subjects included 20% Black/African American participants, a group at increased risk for dementia and usually underrepresented in clinical trials. Although hypotensive episodes were significantly higher in the candesartan group, they were mostly asymptomatic. Candesartan use did not result in an alteration in renal function or potassium levels. The authors showed that treatment was not only safe but also effective. Specifically, candesartan treatment resulted in increased levels of CSF amyloid-β42 and amyloid-β40, while at the same time, the placebo group showed significantly decreased levels of CSF amyloid-β42 and amyloid-β40. There was no effect on whole brain ^11^C-PiB uptake. The regional analysis did show decreased uptake in the parahippocampal region involved in visuospatial tasks and episodic memory, which was not part of their cognitive assessment. However, they have demonstrated that candesartan resulted in significant improvement in the measurement of executive function, measured by Trail Making Test, part B, and a trend for protection was observed in the composite cognitive score. When taking a closer look at the figures, it seems that maximum benefit was achieved at 6 months and the benefits wean at 12 months. This study also evaluated functional network connectivity (FNC). It showed that candesartan was associated with increased FNC between subcortical and auditory network areas and cognitive control and auditory network areas. At the same time, there was decreased FNC between auditory and sensorimotor network areas. Candesartan did not alter default mode network interconnectivity, hippocampal or entorhinal cortex volume, CSF tau levels, or ^18^F-flortaucipir tau imaging levels.

This trial is an important study since it is the first one evaluating the impact of candesartan, an ATR1B, on cognitive function and Alzheimer’s disease biomarkers in participants with MCI without hypertension, and it is the first one to show medication not only reducing beta amyloid in the brain but also improving cognition. By choosing normotensive participants, the study removes the potential confounding role of blood pressure. This study is also markedly different from previous RCTs by its broad availability of both CSF and imaging Alzheimer’s disease biomarkers and detailed cognitive measures, allowing a better understanding of the potential mechanisms in the disease process affected by candesartan. However, we have only been given a partial picture. The authors mainly focus on the amyloid and tau biomarkers and do not address vascular neuropathology. There is evidence from autopsy studies that people with Alzheimer’s disease have combinations of various forms of vascular Alzheimer’s disease and vascular pathology.

Additional pathways need to be explored to better understand the exact mechanisms by which candesartan and other AT1RB exert their potentially beneficial effect in addition to the amyloid pathway.

The brain has its own RAS, which functions independently but interacts with the systemic RAS. Over the last decades, a complex system has been identified with classical and regulatory pathways containing essential peptides and enzymes.^7^ Some of the RAS peptides, angiotensin II, have been associated with increased pro-inflammatory cytokines and decreased anti-inflammatory cytokines in Alzheimer’s disease.^6^ Angiotensin II also increases oxidative stress and neurovascular damage, and ATR1B in animal studies has been shown to counteract these effects.^6^ Thus, measuring these additional pathological markers would add valuable information.

It would also be helpful to evaluate the role of changing levels of RAS components (peptides and enzymes) during Alzheimer’s disease progression and treatment. It has been suggested that there is an imbalance between the internal pathways, the classical and the regulatory RAS, showing a reduced activity of the regulatory RAS pathway, which is highly correlated with worsening Alzheimer’s disease neuropathology.^7^ By understanding the RAS pathways, we could also understand how AT1RB, including candesartan, could alter these pathways and potentially alter the Alzheimer’s disease process.

In conclusion, studies on antihypertensive medication use, such as this study with candesartan, and the potential impact on MCI inform possible mechanisms of action and possibly identify new pharmacological targets of cognitive decline in MCI. This study provides scientific evidence to proceed to larger and longer randomized clinical trials to evaluate whether candesartan could slow or prevent the progression of MCI to Alzheimer’s disease.

## Data Availability

Data sharing is not applicable to this article as no new data were created or analyzed.
